# Oblique Low-Velocity Impact Response and Damage Behavior of Carbon-Epoxy Composite Laminates

**DOI:** 10.3390/ma15155256

**Published:** 2022-07-29

**Authors:** Jin Sun, Linhai Huang, Junhua Zhao

**Affiliations:** Jiangsu Key Laboratory of Advanced Food Manufacturing Equipment and Technology, Jiangnan University, Wuxi 214122, China; junhua.zhao@163.com

**Keywords:** oblique impact, laminates, impact angle, delamination damage

## Abstract

The low-velocity impact behavior of carbon-epoxy cross-ply composites was numerically investigated, examining the effect of impact angle. A plastic continuum damage model, introducing the cohesive interface to describe delamination damage, was established and was validated by available experimental data. Impact histories, progressive deformation, stress transfer, and impact damage are respectively discussed. The results show that an increase in impact angle intensifies the action of tangential force, and gradually transfers energy absorption from normal plastic deformation to tangential deformation and friction, which dissipates more energy through relatively longer contact duration and larger impactor displacement. The delamination damage to upper layers is more affected by tangential loads, intensifying with the increase of the impact angle, and the damage area to the top interface is increased by 132.1% from 0° impact to 60° impact. Meanwhile, the delamination damage to lower layers is mainly determined by normal loads, weakening with the increasing impact angle overall, and the damage area of the lowest interface decreases by 36.6% from 0° impact to 60° impact.

## 1. Introduction

Due to the growing demand for weight reduction of engineering structures, composite laminates have been attracting more and more attention for their application in aerospace, civil engineering, and marine structures, reliant on their high strength, light weight, and excellent energy absorption [1,2,3,4]. However, in practical engineering situations, composite structures are susceptible to low-velocity impacts resulting from falling tools, hailstones, runway sand, etc. In epoxy composite materials, microcracks can be produced and develop due to low shear strain limited by reinforcing material, and their further propagation may cause macroscale damage [5]. Impact-induced damages, especially delamination damage, can cause certain degradations in mechanical properties and potential failures of materials [6,7]. A number of studies have been conducted to focus on the normal impacts on materials. Composite laminates in service are more frequently subjected to low-velocity impacts at certain oblique angles. Hence the evaluation of impact response and damage behavior of composite laminates under oblique impact seems to be necessary.

Many researchers have investigated the behavior of composite laminates under normal impacts. Long et al. [8] reconstructed matrix cracks in three-dimensional space to analyze their distribution and propagation within composite laminates under low-velocity impact. Taking into account strip impactors, Tuo et al. [9] studied the impact damage and residual mechanical properties of composite laminates with the help of DIC and infrared thermography. Liao et al. [10] discussed the effect of laminate thickness on the low-velocity impact responses of Z-pinned laminates with different layup patterns. Falco et al. [11] showed the capability of the virtual testing framework to accurately predict the damage and failure mechanisms in composite laminates subjected to low-velocity impact. Lopresto et al. [12] studied the low-velocity impact behavior of polymeric fiber laminates at room- and in extreme temperature conditions, focusing on the influence of temperature and resin.

Numerical methods are increasingly applied to predict the low-velocity impact behavior of composite laminates due to their high efficiency and low cost, and a reasonable analysis model can improve the accuracy of the computation. The prediction of impact-created irreversible deformation is relatively difficult due to the complexity of the mechanism for its formation. Some polymer matrix composites experience elastic damage deformation before the final collapse, and a number of studies have predicted their elastic damage mechanisms under low-velocity impact using the elastic damage model. However, some other composites have been experimentally confirmed to experience distinct plastic deformation [13,14,15]. It is therefore necessary to introduce a plastic damage model to investigate their low-velocity impact behaviors. By introducing a plastic damage model for intralaminar damage, Liao et al. [16] numerically explored the dynamic mechanical responses and damage mechanisms of plastic fiber-reinforced polymer matrix composite laminate under low-velocity impact, proving that the plastic damage model brought higher precision than the elastic damage model, especially under relatively large impact energy. He et al. [17] proposed a method based on an anisotropic elastoplastic theory to predict permanent indentation due to low-velocity impact on laminated composites, achieving a desirable agreement with the experimental results. Chen et al. [18] proposed a combined elastoplastic damage model for the progressive failure analysis of composite laminates subjected to transverse low-velocity impact, receiving accurate predictions.

The influences of specific conditions such as stacking sequence, impactor shape, and laminate thickness on the impact response have been involved in earlier studies. However, a reduced number of works have focused on the oblique impact behavior of composite structures. Kazemianfar et al. [19] compared the mechanical properties of 2D and 3D woven composites under oblique low-velocity impact. Pascal et al. [20] performed low-velocity normal impacts and medium-velocity oblique impacts on sandwich panels using the finite element method. Ivanez et al. [21] conducted an experimental and numerical study of low-velocity oblique impacts on composite sandwich plates, considering several impact angles and impact energies.

This paper paid attention to the oblique low-velocity impact behavior of cross-ply composites. A plastic continuum damage model was established, employing a cohesive interface to simulate delamination damage. Under given impact angles and energies, the impact histories, progressive deformation, and stress distribution were recorded to illustrate oblique impact response. Surface and delamination damage in certain working conditions were demonstrated to examine the effect of impact angle on impact damage.

## 2. Material Properties and Modeling

To study the effect of oblique angles on the low-velocity impact behavior of cross-ply composite laminates, the component materials and layup pattern are cited in [22], so that the numerical results can be validated by available experimental data. Accordingly, T300/YH69 composite laminates comprising carbon fiber T300 as reinforced fiber and YH69 as epoxy system were employed, following the layup pattern of ${\left[0_{2}/90_{2}\right]}_{4S}$. Each ply had a nominal thickness of 0.12 mm, and the size of each specimen was 80 mm × 80 mm.

### 2.1. Intralaminar Constitutive Model

A large number of experimental studies have shown that since most of the matrix materials have plastic mechanical behaviors, most of the fiber-reinforced resin matrix composite laminates show plastic and nonlinear characteristics under transverse compression and shear loads, especially in the shear direction [23,24,25]. To reflect plastic deformation and damage accumulation during impact, a plastic continuum damage model was built to simulate the progressive failure of laminates. The constitutive equation is expressed by

(1) $$\sigma_{\mathrm{ij}}=C_{ijkl}\varepsilon_{kl}^{e}$$

where $\sigma_{\mathrm{ij}}$, $C_{ijkl}$ and $\varepsilon_{kl}^{e}$(i, j, k, l=1, 2, 3) are the engineering stress, stiffness matrix, and elastic strain components; $\varepsilon =\varepsilon^{e}+\varepsilon^{p}$ and $\varepsilon^{p}$ the Green–Lagrange strain tensor and plastic strain; and subscripts 1, 2, and 3 the fiber direction, direction perpendicular to fiber in plane, and direction out of plane, respectively. The damage degree from no damage to complete failure is characterized by damage variables $d_{I}$. The damage stiffness matrix is given by

(2) $$C_{d}=\frac{1}{\Delta }\left[\begin{array}{cccccc} d_{f}E_{11}\left(1-d_{m}\nu_{23}\nu_{32}\right) & d_{f}d_{m}E_{11}\left(\nu_{21}+\nu_{23}\nu_{31}\right) & d_{f}E_{11}\left(\nu_{31}+d_{m}\nu_{21}\nu_{32}\right) & & & \\ & d_{m}E_{22}\left(1-d_{f}\nu_{13}\nu_{31}\right) & d_{m}E_{22}\left(\nu_{32}+d_{f}\nu_{12}\nu_{31}\right) & & & \\ & & E_{33}\left(1-d_{f}d_{m}\nu_{12}\nu_{21}\right) & & & \\ & & & \Delta d_{f}d_{m}S_{12} & & \\ & & & & \Delta d_{f}d_{m}S_{23} & \\ & & & & & \Delta d_{f}d_{m}S_{13} \end{array}\right]$$

where $d_{f}$ and $d_{m}$ are damage variables of fiber and matrix, obtained by

(3) $$\left\{\begin{array}{c} d_{f}=\left(1-d_{ft}\right)\left(1-d_{fc}\right)\\ d_{m}=\left(1-S_{mt}d_{mt}\right)\left(1-S_{mc}d_{mc}\right)\\ \Delta =1-d_{f}d_{m}\nu_{12}\nu_{21}-d_{m}\nu_{23}\nu_{32}-d_{f}\nu_{13}\nu_{31}-2d_{f}d_{m}\nu_{21}\nu_{32}\nu_{13} \end{array}\right.$$

in which the subscripts *ft*, *fc*, *mt* and *mc* are fiber tension, fiber compression, matrix tension, and matrix compression, respectively.

### 2.2. Plastic Flow Rule

The plastic behavior of laminates is realized by introducing a plastic yield criterion as follows [26,27,28]:

(4) $$F\left(\bar{\sigma },{\tilde{\varepsilon }}^{p}\right)=\bar{\sigma }-\tilde{\sigma }\left({\tilde{\varepsilon }}^{p}\right)=0$$

(5) $$\bar{\sigma }=\sqrt{\frac{3}{2}\left({\bar{\sigma }}_{22}^{2}+{\bar{\sigma }}_{33}^{2}\right)-3{\bar{\sigma }}_{22}{\bar{\sigma }}_{33}+6{\bar{\sigma }}_{23}^{2}+3a_{66}\left({\bar{\sigma }}_{12}^{2}+{\bar{\sigma }}_{13}^{2}\right)}$$

(6) $$\tilde{\sigma }\left({\tilde{\varepsilon }}^{p}\right)=\beta {\left({\tilde{\varepsilon }}^{p}\right)}^{n_{p}}$$

where $\bar{\sigma }$ is the equivalent stress, ${\bar{\sigma }}_{ij}\left(i,j=1,2,3\right)$ the deviatoric stress tensor, ${\tilde{\varepsilon }}^{p}$ the equivalent plastic strain, $\tilde{\sigma }$ the plastic hardening stress, $a_{66}$ the orthotropy parameter, and β and $n_{p}$ the constants to fit the off-axis experimental curve. The equivalent plastic strain rate is obtained by

(7) $$d\varepsilon_{ij}^{p}=\Delta \lambda \frac{\partial F}{\partial {\bar{\sigma }}_{ij}},\;(i,j=1,2,3)$$

where Δλ and $\frac{\partial F}{\partial {\bar{\sigma }}_{ij}}$ are plastic flow factor and plastic gradient, respectively. Combining Equations (4)–(7), one has:

(8) $$\left[\begin{array}{c} d\varepsilon_{11}^{p}\\ d\varepsilon_{22}^{p}\\ d\varepsilon_{33}^{p}\\ d\varepsilon_{12}^{p}\\ d\varepsilon_{23}^{p}\\ d\varepsilon_{13}^{p} \end{array}\right]=\Delta \lambda \frac{\partial F}{\partial {\bar{\sigma }}_{ij}}=\Delta \lambda \left[\begin{array}{c} 0\\ \frac{3}{2}\frac{\left({\bar{\sigma }}_{22}-{\bar{\sigma }}_{33}\right)}{\bar{\sigma }}\\ \frac{3}{2}\frac{\left({\bar{\sigma }}_{33}-{\bar{\sigma }}_{22}\right)}{\bar{\sigma }}\\ \frac{3a_{66}{\bar{\sigma }}_{12}}{\bar{\sigma }}\\ \frac{6{\bar{\sigma }}_{23}}{\bar{\sigma }}\\ \frac{3a_{66}{\bar{\sigma }}_{13}}{\bar{\sigma }} \end{array}\right]$$

### 2.3. Damage Initiation and Evolution

The initiation of damage to laminates is identified by strain-expressed Hashin criteria as follows:

Fiber tensile failure ($\varepsilon_{11}\geq 0$)

(9) $$F_{ft}={\left(\frac{\varepsilon_{11}}{\varepsilon_{ft}^{0}}\right)}^{2}\geq 1,\;\varepsilon_{ft}^{0}=\frac{X^{T}}{E_{11}}$$

Fiber compressive failure ($\varepsilon_{11}<0$)

(10) $$F_{fc}={\left(\frac{\varepsilon_{11}}{\varepsilon_{fc}^{0}}\right)}^{2}\geq 1,\;\varepsilon_{fc}^{0}=\frac{X^{c}}{E_{11}}$$

Matrix tensile failure ($\varepsilon_{22}+\varepsilon_{33}\geq 0$)

(11) $$F_{mt}={\left(\frac{\varepsilon_{22}}{\varepsilon_{mt}^{0}}\right)}^{2}\geq 1,\;\varepsilon_{mt}^{0}=\frac{Y^{T}}{E_{22}}$$

Matrix compressive failure ($\varepsilon_{22}+\varepsilon_{33}<0$)

(12) $$F_{mc}={\left(\frac{E_{22}\varepsilon_{22}+E_{33}\varepsilon_{33}}{2S_{12}}\right)}^{2}+\frac{\varepsilon_{22}+\varepsilon_{33}}{\varepsilon_{mc}^{0}}\left[{\left(\frac{E_{22}\varepsilon_{mc}^{0}}{2S_{12}}\right)}^{2}-1\right]+{\left(\frac{G_{23}}{S_{23}}\right)}^{2}(\varepsilon_{23}^{2}-\frac{E_{22}E_{33}}{G_{23}}\varepsilon_{22}\varepsilon_{33})+{\left(\frac{G_{12}\varepsilon_{12}}{S_{12}}\right)}^{2}+{\left(\frac{G_{13}\varepsilon_{13}}{S_{13}}\right)}^{2}\geq 1\;\varepsilon_{mc}^{0}=\frac{Y^{C}}{E_{22}}$$

where $\varepsilon_{ij}$ and $E_{ij}(i,j=1,2,3)$ are the elastic strain tensor and Young’s modulus, $\varepsilon_{I}^{0}(I=ft,fc,mt,mc)$ the initial failure strain, $X^{T}$ and $X^{C}$ the tensile and compressive strength in the axial direction of the fiber, $Y^{T}$ and $Y^{C}$ the tensile and compressive strength in the transverse direction of the fiber, and $S_{ij}$ the shear strength.

**Figure 1:**
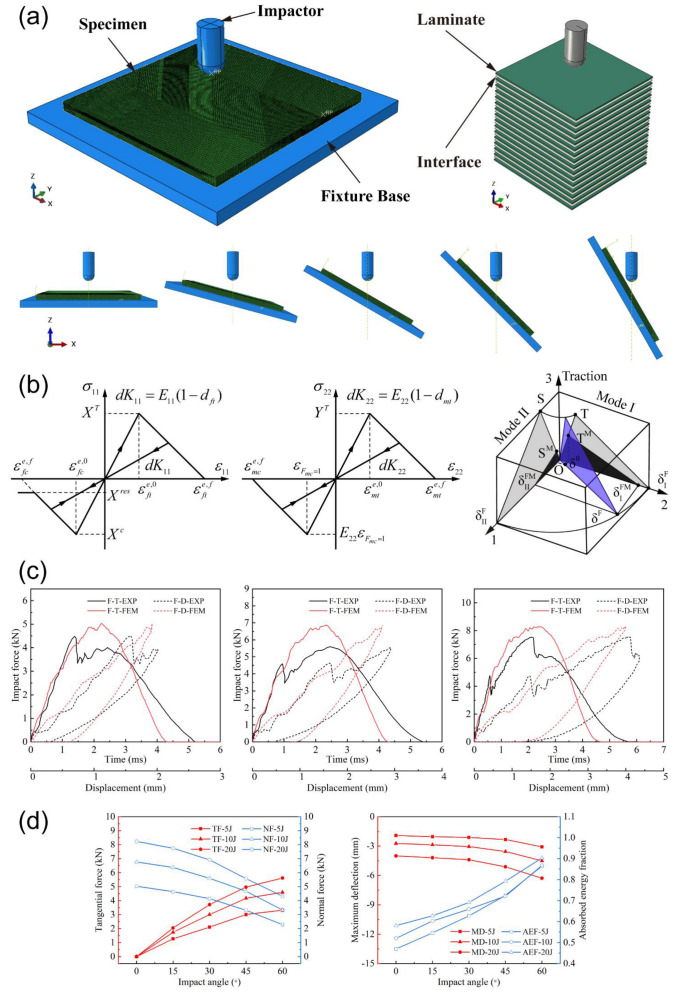
Numerical simulation of oblique impact: (**a**) impact model with different impact angles, (**b**) intralaminar stress–strain relationship and cohesive zone model under mixed-mode loading, (**c**) validation of numerical model, and (**d**) overall numerical results of impact response.

After the initiation of damage, a damage evolution model based on fracture energy was employed for performance degradation. The damage variables are given by

(13) $$d_{I}=\frac{\varepsilon_{I}^{f}}{\varepsilon_{I}^{f}-\varepsilon_{I}^{0}}(1-\frac{\varepsilon_{I}^{0}}{\varepsilon_{I}})\;\left(d_{I}\in (0,1),I=ft,fc,mt,mc\right)$$

where $\varepsilon_{I}^{f}$ is final failure strain. When the damage variable reaches one, the strain is obtained by

(14) $$\varepsilon_{ft}^{f}=\frac{2G_{ft}}{X^{T}l}$$

(15) $$\varepsilon_{fc}^{f}=\frac{2G_{fc}}{X^{C}l}$$

(16) $$\varepsilon_{mt}^{f}=\frac{2G_{mt}}{Y^{T}l}$$

(17) $$\varepsilon_{mc}^{f}=\frac{2G_{mc}}{Y^{C}l}$$

where $G_{I}(I=ft,fc,mt,mc)$ is the fracture energy density, and l the characteristic length of element. The fiber stress–strain relationship in the axial/transverse directions is shown in Figure 1b.

### 2.4. Interlaminar Properties

The interlaminar interface, composed of cohesive elements, is built to simulate delamination damage. The damage initiation is identified by a quadratic failure criterion as follows:

(18) $$\frac{{\langle t_{n}\rangle }^{2}}{N^{2}}+\frac{t_{s}^{2}}{S^{2}}+\frac{t_{t}^{2}}{T^{2}}=1$$

where $\langle \cdot \rangle$ is the Macauley bracket, $t_{n}$, $t_{s}$, and $t_{t}$ the normal and shear tractions, *N*, *S*, and *T* the normal and shear interface strengths. After damage initiation, damage evolution is governed by the B-K criterion [29]. Under mixed modes (see Figure 1b), the dissipated energy is calculated by

(19) $$G^{C}=G_{n}^{C}+(G_{s}^{C}-G_{n}^{C}){\left\{\frac{G_{S}}{G_{T}}\right\}}^{\eta }$$

where $G_{n}^{C}$ and $G_{s}^{C}$ are the interlaminar fracture energies in normal and shear directions, $G_{T}$ and $G_{S}$ the total and out-of-plane dissipated energies, and η the material coefficient.

## 3. Finite Element Model

To simulate oblique low-velocity impact, the impact angle (defined as the angle between the normal direction of the laminate and impact direction) was respectively set to 0°, 15°, 30°, 45°, and 60°, as shown in Figure 1a. The hemispherical impactor with a diameter of 10 mm was considered as an analytical rigid body. The concentrated mass of 5.61 kg and initial velocity of 1.34, 1.89, and 2.67 m/s along the z-direction were assigned to the impactor to achieve 5, 10, and 20 J impact energies. The impactor only releases the translational constraint along the z-direction, and the specimen constrains all the translational degrees of freedom. The general contact in ABAQUS is applied to reflect contact behavior in the model. The material properties of lamina and interface are shown in Table 1.

The rigid body element R3 D4 was used for the impactor, and the bilinear cohesive element COH3 D8 with finite thickness of 0.001 mm was used for the interface. The reduced integration element C3 D8 R was used for the lamina, and the stiffness control method was employed to avoid the hourglass effect. In consideration of both computational efficiency and accuracy, the mesh is refined in impact area.

## 4. Results and Discussion

### 4.1. Validation of the Numerical Model

To ensure the effectiveness of the simulation, the impact responses, including force-time and force-displacement relations with 0° impact angle under 5 J, 10 J, and 20 J energies, were first validated by available experimental data in [22], and a good consistency was obtained, as shown in Figure 1c. The peak force, maximum displacement of impactor, and absorbed energy obtained by simulation and experiment are compared in Table 2. The maximum error is within 20%, which proves that the numerical model is reliable for further analysis.

### 4.2. Impact Response

The total trends of tangential/normal force, maximum deflection, and energy absorption rate with the increasing impact angle under three energies are demonstrated in Figure 1d, and the detailed calculation results will be discussed as follows.

**Figure 2:**
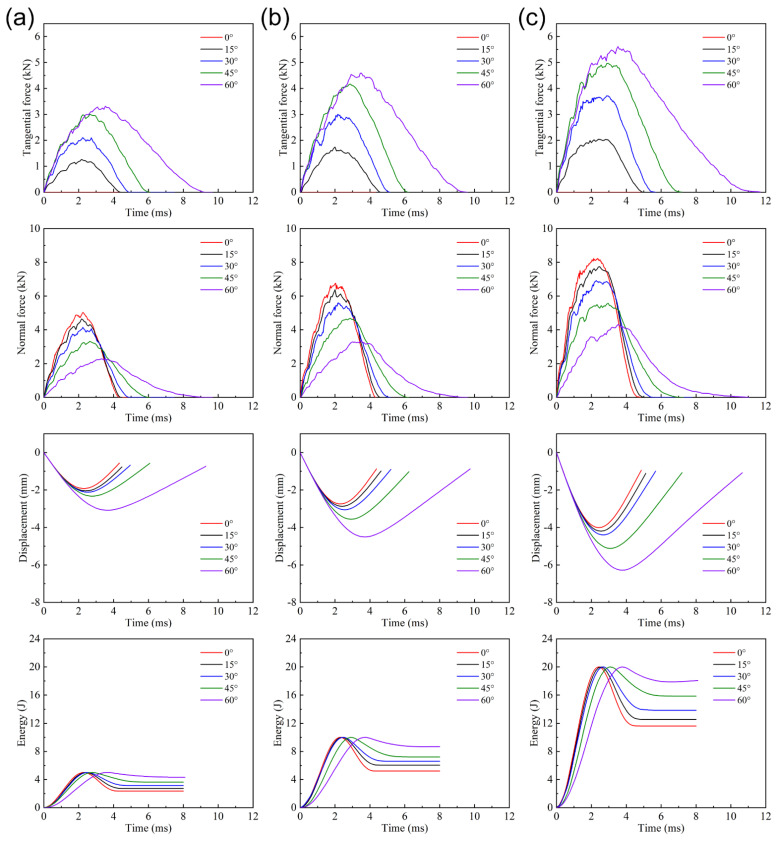
Impact responses with different impact angles under (**a**) 5 J, (**b**) 10 J, and (**c**) 20 J impact energies.

Figure 2 describes the tangential/normal force-time, displacement-time and energy-time histories with 0°, 15°, 30°, 45°, and 60° impact angles under 5 J, 10 J, and 20 J energies. The tangential/normal peak force, maximum displacement of impactor, and absorbed energy in each working condition are shown in Table 3. As the impact angle grows, the contact duration, tangential force component, and impactor displacement increase, while the normal force component declines. Particularly, the tangential force under 0° impact angle always remains at zero. The larger impact angle brings the impactor more displacement by longer contact duration and causes the tangential/normal peak force to appear later. The increase in impact angle transfers the energy absorption from plastic deformation to friction and dissipates more energy, experiencing relatively longer duration and larger impactor displacement. Under 5 J, 10 J, and 20 J impact energies, the energy absorption rates of 0° impact are 47.0%, 52.0%, and 58.1%, while those of 60° impact are 86.6%, 86.8%, and 90.4%. In addition, the growth of impact energy improves the whole level of tangential/normal force, impactor displacement, and absorbed energy.

**Figure 3:**
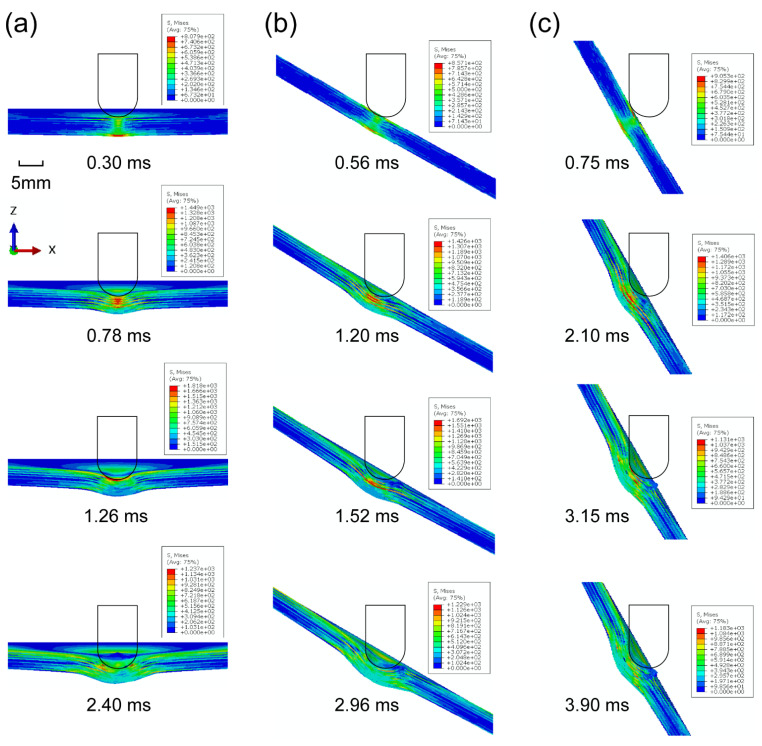
Progressive deformation and stress distribution of laminates with (**a**) 0°, (**b**) 30°, and (**c**) 60° impact angles under 20 J impact energy.

**Figure 4:**
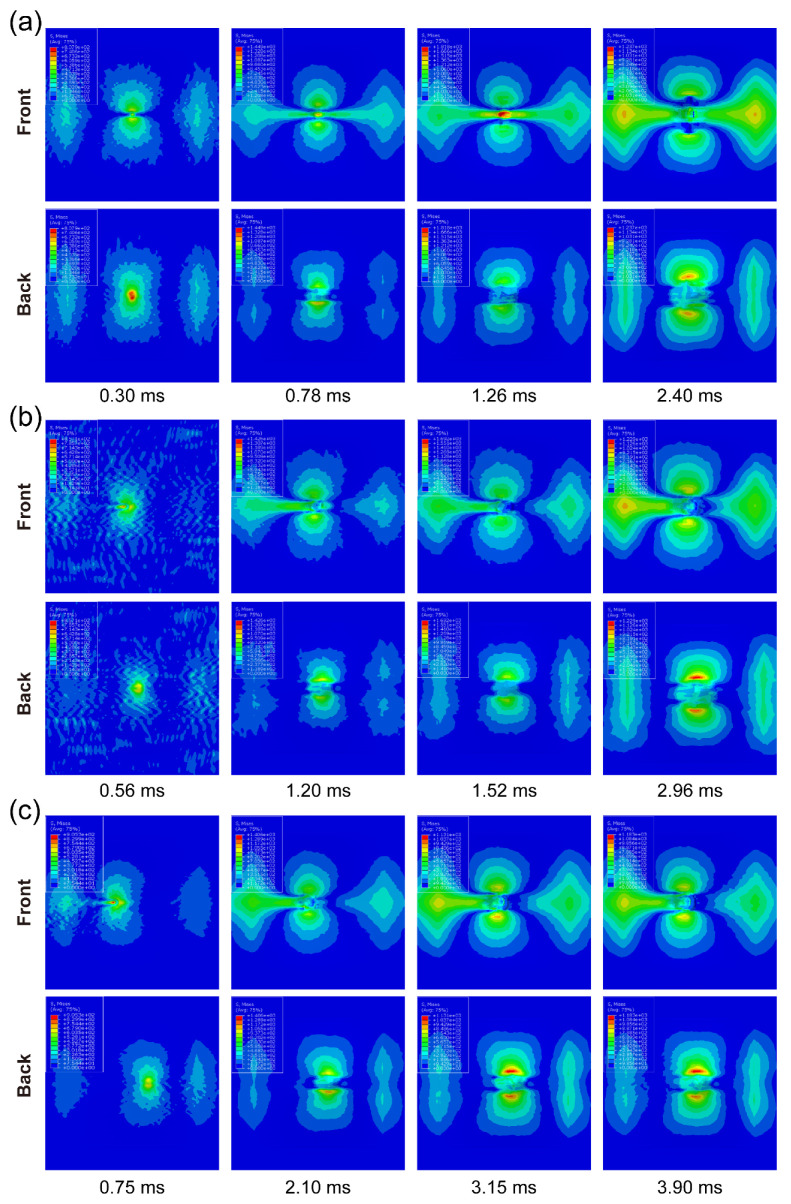
Progressive stress distribution on both sides of laminate with (**a**) 0°, (**b**) 30°, and (**c**) 60° impact angles under 20 J impact energy.

Figure 3 shows the progressive deformation and stress distribution of the laminate cross-section with 0°, 30°, and 60° impact angles under 20 J energy. For all cases, the stress was first distributed in an H-shape covering the whole thickness of the laminate, which indicates that the impact loading first spreads on both sides, namely the compressed top side and stretched bottom side. The stress concentration firstly appears at the bottom, and then transfers upwards as the stress of the outer layer propagates outwards from the impacted area. With the further outward transfer of inner-layer stress, the degree of stress concentration declines, and the stress is distributed more evenly. The deformation and stress distribution under 0° impact are nearly symmetrical, while those under oblique impact are asymmetrical. The larger the impact angle, the weaker the normal squeeze, and the more severe the tangential friction; thus not only normal plastic deformation but also tangential deformation are produced. It is seen from Table 3 that the dent depth shows a downward trend as the impact angle rises. It should be noted that although the increase in impact angle decreases the normal force component, limiting the normal deformation, it also raises the impactor displacement and contact duration, which can increase the normal deformation to a certain extent. Especially at small impact angles, this competition is evident, hence the dent depth at small impact angles can even be deeper than that under normal impact (e.g., in the conditions of 15°/20 J and 30°/20 J). Figure 4 depicts the corresponding stress propagation processes on the top and bottom sides, under the same working conditions as in Figure 3. The stress is first concentrated in the impacted areas on both sides, and then propagates outward, distributed more widely and more evenly. As the impact angle grows, the stress distribution becomes asymmetrical, since the initial contact area of the impactor deviates from the center of the laminate, and the load is no longer symmetrical either.

### 4.3. Impact Damage

**Figure 5:**
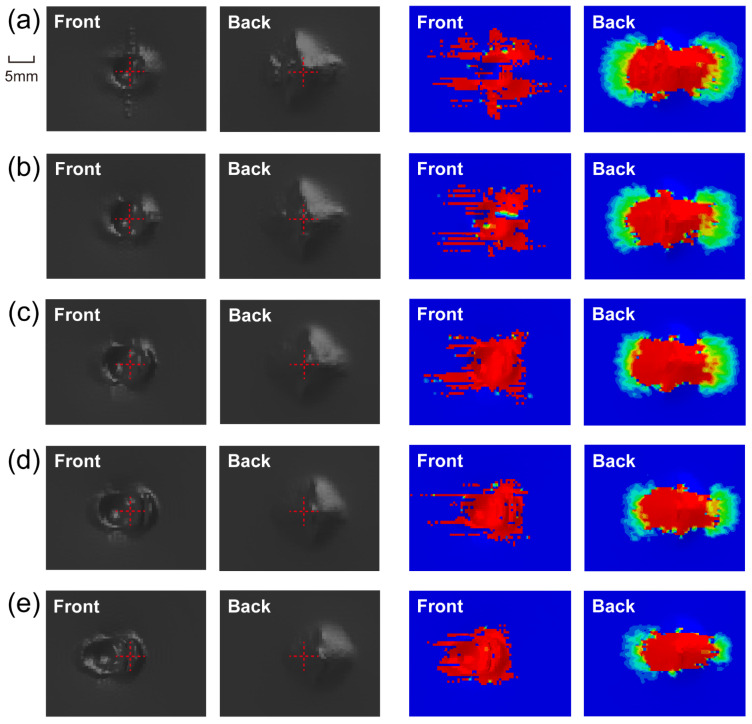
Damage morphology and matrix damage on both sides of laminate with (**a**) 0°, (**b**) 15°, (**c**) 30°, (**d**) 45°, and (**e**) 60° impact angles under 20 J impact energy.

**Figure 6:**
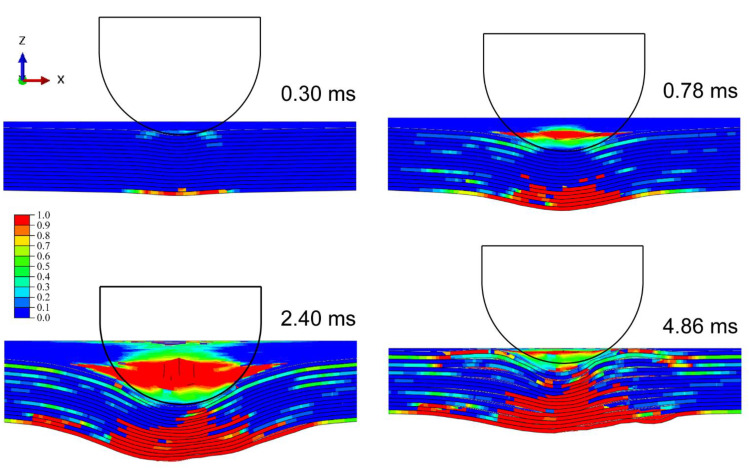
Evolution of matrix tensile damage with 0° impact angles under 20 J impact energy.

**Figure 7:**
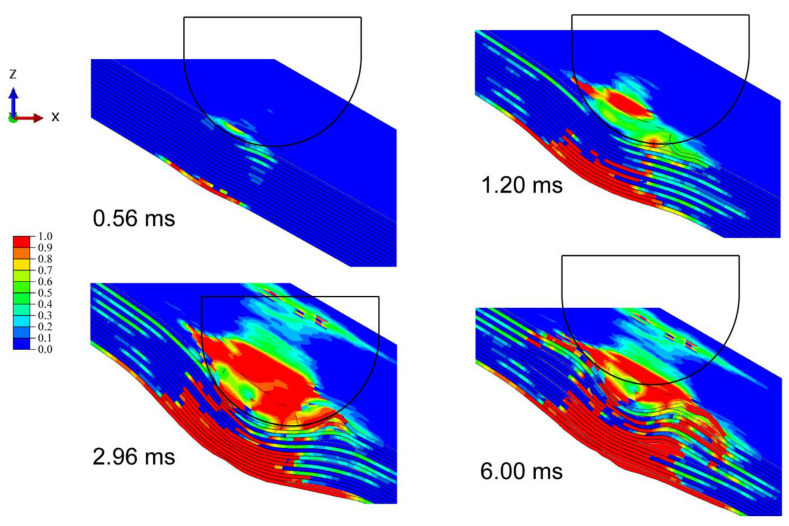
Evolution of matrix tensile damage with 30° impact angles under 20 J impact energy.

**Figure 8:**
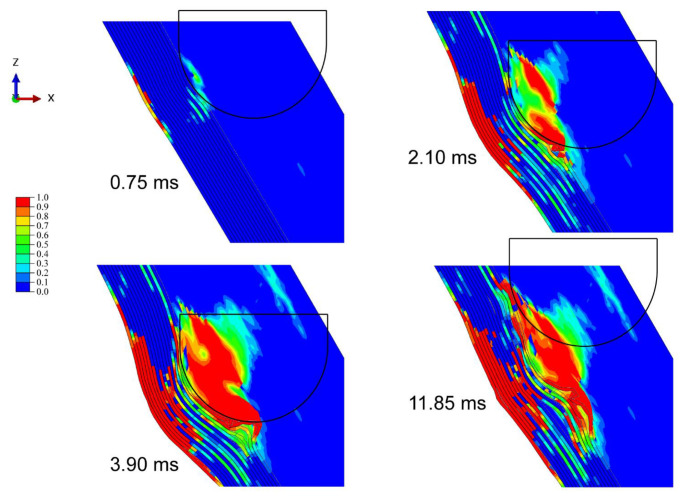
Evolution of matrix tensile damage with 60° impact angles under 20 J impact energy.

Figure 5 shows the damage morphology and matrix compressive/tensile damage on the front/back side under each impact angle and energy. The hemispheric impactor leaves a circular dent in the front side under normal impact, while creating elliptical ones under oblique impact. The greater the impact angle, the longer the major axis of the ellipse and the distance between the centers of dent and laminate. Normal impact mainly produces normal plastic deformation by providing only normal force, whereas oblique impact brings tangential deformation by offering a tangential force component, causing a certain slippage of the impactor on the impacted surface, which contributes to greater impactor displacement and contact duration. The increase in impact angle enlarges the deviation distance between the initial contact location of the impact and the laminate center and more easily produces impactor slippage. The damage morphology of the front side is attributed to the contact between the impactor and the laminate, and the displayed matrix damage reflects the growth of the impactor’s slide with the increasing impact angle. Additionally, the impact causes cruciform bumps on the back side, which are consistent with the stacking angle of the laminate. The damage area on the back side is produced by bending stress due to the normal impact loads, hence it declines as the impact angle increases. Taking matrix tensile damage as an example, Figure 6, Figure 7 and Figure 8 describe the damage evolution of laminates with 0°, 30°, and 60° impact angles under 20 J impact energy, respectively. For all the working cases, the damage always initiates at the bottom, and propagates upwards and outwards. In contrast, 0° impact produces the largest normal deformation, leaving relatively severe delamination at the bottom. As the impact angle grows, the squeeze of the surface layer along the tangential direction becomes more serious, producing obvious delamination in the upper layers. The quantitative analysis of delamination damage will be discussed below.

**Figure 9:**
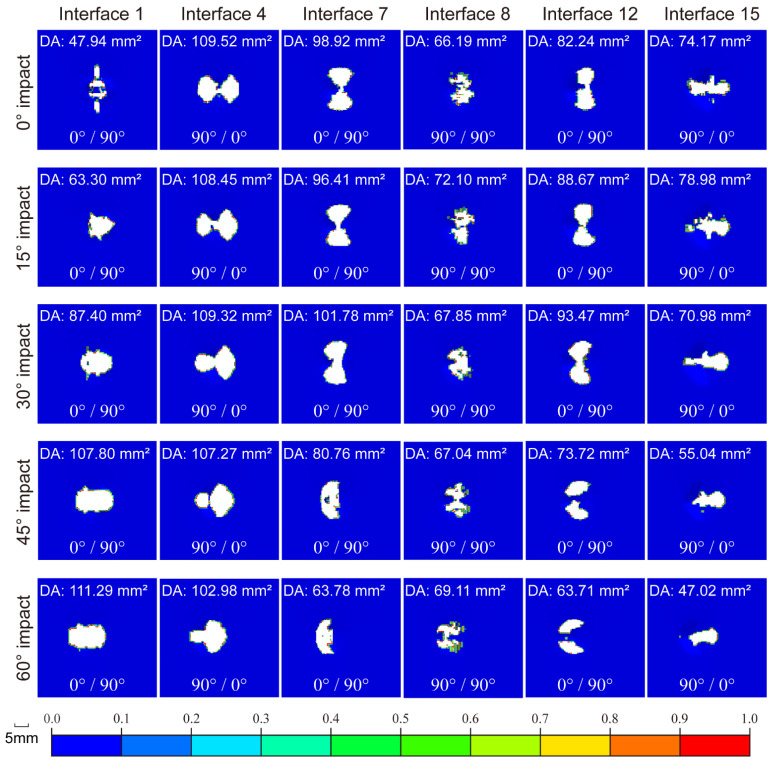
Delamination damage in given interfaces under 20 J impact energy.

Table 3 shows the maximum length, width, and area of delamination damage, superimposing all the interfaces. As an example to illustrate the effect of impact angle on delamination damage, Figure 9 demonstrates the delamination damage in given interfaces with different impact angles under 20 J energy, where the damage area (DA) and the stacking angles of adjacent layers on both sides of each interface are marked. At a 0° impact angle, the delamination expansion direction of each interface is consistent with the stacking angle of its lower layer, and the delamination damage between the adjacent layers with the same stacking angle (e.g., interface 8) is relatively weak. This phenomenon agrees well with [30]. As the impact angle increases, the influence of tangential force becomes more and more significant, and the delamination damage begins to extend along the tangential direction, especially for the upper interfaces (e.g., interfaces 1 and 4). However, this influence is simultaneously weakened downward. Interface 15 is weakly affected by the tangential force, and its delamination area decreases with the increaser in impact angle, which can be attributed to the effect of normal force.

**Figure 10:**
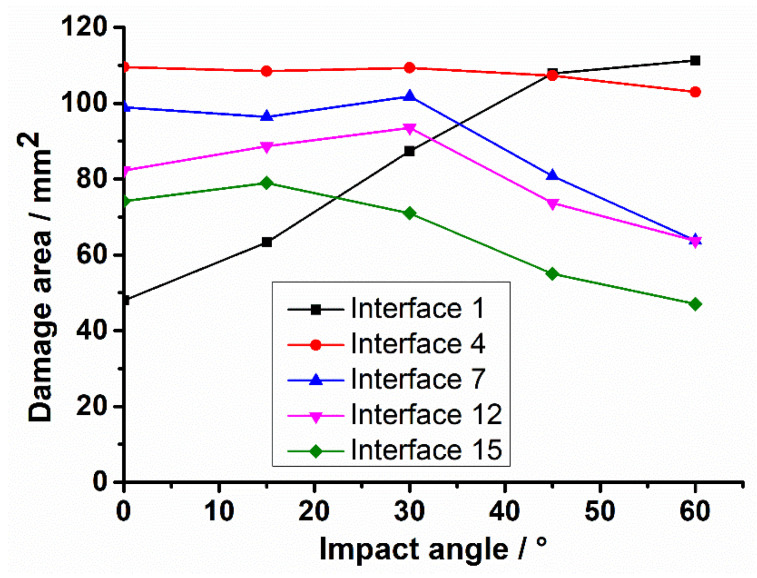
Variation tendency of damage area with impact angle under 20 J impact energy.

As the impact angle grows, the tangential force rises while the normal force drops (See Figure 2), and their contributions to delamination damage may correspondingly increase and decrease to some extent. The competition will determine whether the damage intensifies or weakens, which means the trend of damage development with impact angle can reflect which force plays the dominant role. Figure 10 shows the interaction of delamination damage area with impact angle, where the interfaces between the adjacent layers with different stacking angles, namely interfaces 1, 4, 7, 12, and 15, are considered. With the increase in impact angle, the damage area of interface 1 always goes up, and is increased by 132.1% from 0° impact to 60° impact, which is more attributed to the influence of tangential force. The curve of interface 4 basically remains at a stable level, because the promoting effect of growing tangential force on damage is declining, and just counteracts the weakening effect of decreasing normal force on damage. For the middle and lower interfaces 7, 12, and 15, the contribution of tangential force to damage is getting smaller, and their damages are gradually governed by normal force, hence their damage areas show varying degrees of decline. The damage area of the lowest interface is decreased by 36.6% from 0° impact to 60° impact. In conclusion, the delamination damage of the upper layers is mainly affected by the tangential loads and intensifies as the impact angle increases. On the other hand, the delamination damage of the lower layers is more determined by the normal loads and weakens with the growing impact angle as a whole.

## 5. Conclusions

Oblique low-velocity impact response and damage behaviors of carbon-epoxy cross-ply composites were numerically studied based on a plastic continuum damage model. The results show that as the impact angle grows, the tangential force rises while the normal force declines. The main form of energy absorption is transferred from normal plastic deformation to tangential deformation and friction, dissipating more energy due to longer contact duration and larger impactor displacement. Under 5 J, 10 J, and 20 J energies, the energy absorption rates of normal impact are 47.0%, 52.0%, and 58.1%, while those of 60° impact are 86.6%, 86.8%, and 90.4%. It was found that the tangential loads primarily affect the delamination damage to upper layers, which gets more severe as the impact angle grows. From 0° impact to 60° impact, the damage area of the top interface is improved by 132.1%. Additionally, the delamination damage to the lower layers was more governed by the normal loads and weakened with the increasing impact angle as a whole. The damage area of the lowest interface decreased by 36.6% from 0° impact to 60° impact.

## Figures and Tables

**Table 1:** Material properties of composite lamina and interface [22].

Lamina	*E*_11_ = 130 GPa, *E*_22_ = *E*_33_ = 7.1 GPa, *G*_12_ = *G*_13_ = 3.6 GPa, *G*_23_ = 3.08 GPa
	*ν*_12_ = *ν*_13_ = 0.32, *ν*_23_ = 0.52, *ρ* = 1600 kg/m^3^
	*X*^T^ = 1760 MPa, *X*^C^ = 1100 MPa, *Y*^T^ = 51 MPa, *Y*^C^ = 167 MPa
	*S*_12_ = *S*_13_ = 70 MPa, *S*_23_ = 60 MPa
	*G*_ft_ = 56 N/mm, *G*_fc_ = 10 N/mm, *G*_mt_ = 0.25 N/mm, *G*_mc_ = 0.75 N/mm
	*a*_66_ = 1.25, *β* = 567.9092, *n*_p_ =0.272405
Interface	*N* = 60 MPa, *S* = *T* = 80 MPa, *η* = 1.45
	*G*_n_ = 0.35 N/mm, *G*_S_ = *G*_T_ = 1.45 N/mm

**Table 2:** Comparison of predictive and experimental data.

Sample	*F*_max_ (kN)		Error (%)	*Dis*_max_ (mm)		Error (%)	*E*_abs_ (J)		Error (%)
	Exp.	FEM		Exp.	FEM		Exp.	FEM	
0°/5 J	4.52	5.03	11.3	2.01	1.92	−4.5	2.41	2.35	−2.5
0°/10 J	5.63	6.67	18.5	2.92	2.74	−6.2	4.86	5.20	7.0
0°/20 J	7.56	8.23	8.9	4.35	4.01	−7.8	13.02	11.62	−10.8

*F*_max_ is peak force, *Dis*_max_ is maximum displacement of impactor, and *E*_abs_ is absorbed energy.

**Table 3:** Statistics of low-velocity impact numerical results.

Sample	*L*_d_ (mm)	*W*_d_ (mm)	*S*_d_ (mm^2^)	*D*_d_ (mm)	*Dis*_max_ (mm)	*F*_t-max_ (kN)	*F*_n-max_ (kN)	*E*_abs_ (J)
0°/5 J	11.09	10.42	71.27	0.63	1.92	0.00	5.03	2.35
15°/5 J	9.37	10.17	70.34	0.62	2.05	1.27	4.64	2.73
30°/5 J	10.23	9.12	66.10	0.54	2.12	2.11	4.14	3.14
45°/5 J	9.80	8.26	63.26	0.39	2.33	3.01	3.31	3.62
60°/5 J	9.98	8.51	55.06	0.21	3.08	3.32	2.28	4.33
0°/10 J	13.07	15.47	115.26	1.06	2.74	0.00	6.67	5.20
15°/10 J	11.96	13.44	109.91	1.04	2.87	1.74	6.37	6.03
30°/10 J	13.44	12.20	111.48	0.90	3.05	3.00	5.60	6.59
45°/10 J	12.20	10.66	97.12	0.67	3.56	4.17	4.67	7.22
60°/10 J	13.50	10.85	99.38	0.46	4.51	4.59	3.33	8.68
0°/20 J	17.38	19.11	213.80	1.34	4.01	0.00	8.23	11.62
15°/20 J	16.58	14.79	202.10	1.45	4.19	2.04	7.74	12.55
30°/20 J	16.95	14.51	201.16	1.40	4.40	3.72	6.91	13.83
45°/20 J	14.98	13.50	168.30	1.17	5.12	4.96	5.57	15.84
60°/20 J	15.47	12.82	158.92	0.82	6.28	5.62	4.31	18.07

*L*_d_ is maximum length of superimposed delamination damage, *W*_d_ is maximum width of superimposed delamination damage, *S*_d_ is area of superimposed delamination damage, *D*_d_ is dent depth, *Dis*_max_ is maximum displacement of impactor, *F*_t-max_ is tangential peak force, *F*_n-max_ is normal peak force, and *E*_abs_ is absorbed energy.
